# Significance and Diagnostic Role of Antimicrobial Cathelicidins (LL-37) Peptides in Oral Health

**DOI:** 10.3390/biom7040080

**Published:** 2017-12-05

**Authors:** Zohaib Khurshid, Mustafa Naseem, Faris Yahya I. Asiri, Maria Mali, Rabia Sannam Khan, Haafsa Arshad Sahibzada, Muhammad Sohail Zafar, Syed Faraz Moin, Erum Khan

**Affiliations:** 1 College of Dentistry, King Faisal University, Al-Ahsa 31982, Saudi Arabia; drzohaibkhurshid@gmail.com; 2 Department of Preventive Dentistry, College of Dentistry, Dar-Al-Uloom University, Riyadh 13314, Saudi Arabia; m.naseem@dau.edu.sa; 3 Department of Preventive Dental Sciences, College of Dentistry, King Faisal University, Al-Ahsa 312982, Saudi Arabia; fasiri@kfu.edu.sa; 4 Department of Orthodontics, Islamic International Dental College, Ripah International University, Islamabad 44000, Pakistan; drmariahmali@gmail.com; 5 Department of Oral Pathology, College of Dentistry, Baqai University, Super Highway, P.O. Box 2407, Karachi 74600, Pakistan; rabia.sannam@baqai.edu.pk; 6 Department of Oral Medicine, Islamabad Dental Hospital (IDH), Islamabad 74700, Pakistan; Haafsa.OralMed16@iideas.edu.pk; 7 Department of Dental Materials, College of Dentistry, Taibah University, Madinah Munawwarah 41311, Saudi Arabia; 8 Department of Dental Materials, Islamic International Dental College, Riphah International University, Islamabad 44000, Pakistan; 9 National Center for Proteomics, Karachi University, Karachi 75270, Pakistan; farazmoin@hotmail.com; 10 Department of Oral Pathology, Institute of Dentistry, Liaquat University of Medical and Health Sciences, Jamshoro 71000, Pakistan; erumkhan_2010@icloud.com

**Keywords:** proteins, antimicrobial peptides, drug, diagnosis, cathelicidins, oral health

## Abstract

Cathelicidins are a group of oral antimicrobial peptides that play multiple vital roles in the human body, such as their antimicrobial (broad spectrum) role against oral microbes, wound healing, and angiogenesis, with recent evidences about their role in cancer regulation. Cathelicidins are present in humans and other mammals as well. By complex interactions with the microenvironment, it results in pro-inflammatory effects. Many in vitro and in vivo experiments have been conducted to ultimately conclude that these unique peptides play an essential role in innate immunity. Peptides are released in the precursor form (defensins), which after cleavage results in cathelicidins formation. Living in the era where the major focus is on non-invasive and nanotechnology, this ultimately leads to further advancements in the field of salivaomics. Based on current spotlight innovations, we have highlighted the biochemistry, mode of action, and the importance of cathelicidins in the oral cavity.

## 1. Introduction

The human body is exposed to harsh environmental conditions and various infectious diseases. Infectious diseases are the global cause of mortality and morbidity. The host’s immune system plays a vital role in protection. There are various levels of immunity such as innate factor, adaptive immunity, and anatomical and physiological barriers. Innate immunity can be further categorized into humoral immunity and cellular mechanisms. In addition, a part of innate factor is build up from a broad category of antimicrobial peptides (AMPs). In the past decades, various innovations and research methods have used antimicrobial peptides as a tool to combat against intruding pathogens, and, hence, are renowned as natural antibiotics [1]. The in vitro experiments using AMPs displayed a wide range of antimicrobial activity including antibacterial, antifungal, and antiviral activity against the offending organism [2]. These peptides provide defence against the offending organism at the initial stages. Recently, AMPs have been shown to have wound healing potential, alteration potential of the host gene expression, and the ability to induce cytokines production, all of which fall under the category of immunomodulatory function. This has been described as their indirect role in the defence [2,3]. The immunomodulatory properties of human AMP are to reduce the level of inflammatory cytokines, help in wound healing, leukocytes activation, and macrophage differentiation [4]. AMPs are found in both prokaryotes and eukaryotes. In mammals, two primary groups of AMPs have been recognised: defensins and cathelicidins. Among various types of antimicrobial peptides, this review is focusing on cathelicidins antimicrobial peptides.

**Figure 1:**
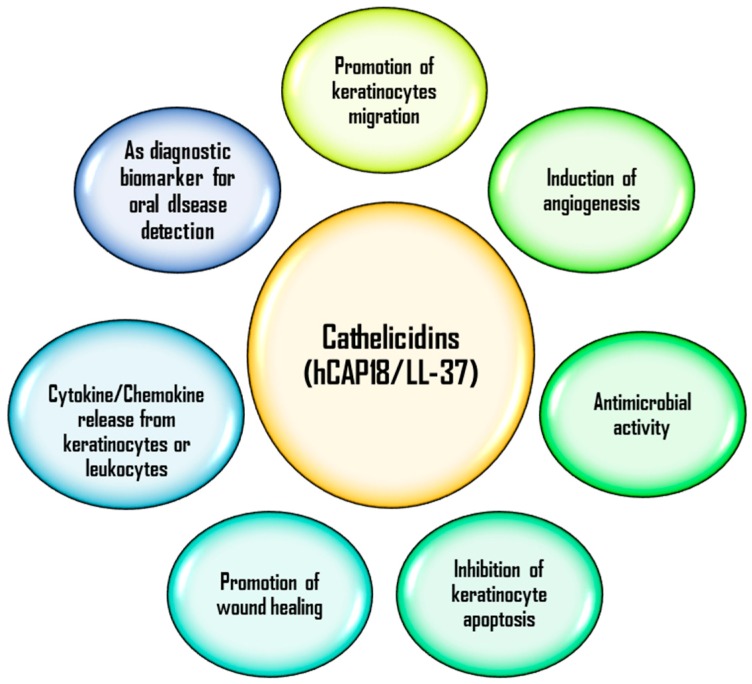
Different functions of human cathelicidins (LL-37) peptides in the human body [4]. hCAP18: human cationic antimicrobial peptide 18 precursor protein.

Cathelicidins (LL-37) is an antimicrobial peptide (Mw ~ 18 kDa) that belong to the cationic amphipathic family found in both mammals (such as rabbits, cattle, horses, pigs, rats, rodents, and ungulates) and non-mammals (such as hagfish, chickens, and salmon) [5,6,7,8]. In mammals, LL-37 are produced by various cells, including skin epithelial cells, leukocytes [9], B-cells, keratinocytes, melanocytes [10], neutrophils, bone marrow cells [11], breast milk [12], mast cells, seminal plasma [13], salivary glands [14], inflamed gingival tissues, and respiratory epithelium [15]. Hence, cathelicidins are among the first defence peptides that come in contact with foreign pathogens and aids in first line defence. Various functions of human LL-37 peptides in the maintenance of human health reference are illustrated in Figure 1.

Historically, following the discovery of Bac5 and seven antimicrobial peptides from bovine neutrophils (in the 1980s), cathelicidins were isolated from pig’s intestine and were classified as mammalian cecropin [10]. In 1995, the human cathelicidins (CAP-18) were isolated from neutrophils [16]. Later on, it was isolated from a variety of human cells, body fluids, and tissues [17]. We have highlighted the biochemistry, mode of action, and the importance of cathelicidins. The antibacterial property of cathelicidins peptides is due to its ability to destabilize the bacterial cell membrane through interactions with proteins and non-polar components of bacterial membranes. Therefore, cathelicidins are an active part of humoral immunity and play a significant role in the direct defence and activation of the inflammatory cells. The role of these peptides is not limited to the antibacterial activity, but its antiviral and anti-fungal actions makes it a biomolecule of greater interest.

## 2. Types and Biochemistry of LL-37

Cathelicidins and their precursor molecules are synthesized after proteolytic cleavage [17]. Based on their structures and molecular weight diversities, these peptides are characterized and are found in a variety of species. The particular gene that is reported to be responsible for the synthesis of cathelicidins in mammals is organized as 4 exons and 3 introns. Four other different genes (*CATH1*, *CATH2*, *CATH3* and *CATH-B1*) have been reported in birds that have structure similar to mammalian peptides. Three genes (*CATH1*, *CATH2*, *CATH3*) encodes for the major part of cathelin-like domain, signal peptides and 5′ untranslated terminal. While the fourth exon (*CATH-B1*) encodes for mature peptides and 3′ untranslated terminals [18].

Cathelicidins are generated as inactive precursor molecule comprises of three parts [8];

(1) N-terminal that is composed of 29–30 amino acid molecules and is assumed to guide the liberation of biologically active peptides;
(2) cathelin-domain comprising of 98–114 amino acid molecules with its function not yet examined; and
(3) C-terminal that comprises of 12–100 amino acid molecules as an active peptide with wide range of antimicrobial property against bacteria, viruses, and fungi.

The reported members of cathelicidins family include;

(1) LL-37 (leucine-leucine 37) that is found in humans [19],
(2) CRAMP (cathelicidins related antimicrobial peptide) found in rats and mice [16],
(3) Flowlicidin 1,2,3 and cathelicidins β-1 found in chickens [20],
(4) CATH-1 and CATH-2 both are found in the Atlantic salmon [21],
(5) p15s found in rodents, and CAP18 in rabbits [22], and
(6) CAP11 is found in guinea pigs and LL-37 in rhesus monkeys [23].

In humans, the gene encoding for the LL-37 is found on chromosome number 3, it is generated by the cleavage of hCAP18 (human cationic antimicrobial peptide 18 precursor protein). The cleavage of the C-terminus end of this precursor protein leads to the formation of LL-37 which is cationic in nature. Both the precursor and the active product are found in a number of human cells and body fluids (human wound fluid, saliva, gingival crevicular fluid (GCF), seminal plasma, vernix, and tracheal aspirates) [14,24,25]. The production of cathelicidin can be regulated by variable number of cytokines, growth factors and activated vitamin D, which constitute the endogenous products [26]. This is important, as vitamin D is known to induce LL-37 in oral epithelial. Moreover, LL-37 also plays an important role in the maintenance of oral health. Presence of LL-37 in saliva and GCF has the potential role of antimicrobial activity against gram negative and gram positive bacteria in the oral cavity and an immunomodulatory function. LL-37 expression has a role in the protection of tooth structures and oral mucosa [27].

## 3. Mechanism of Action against Microbes

**Figure 2:**
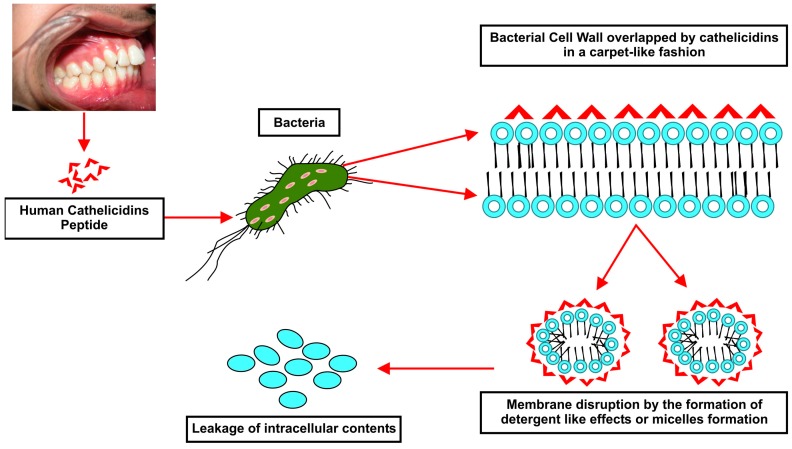
Illustration representing the cathelicidins mechanism of action against microbes specifically for LL-37 and oral cavity.

Antimicrobial peptides are components of host defence proteins that act against the microbial invasion by various mechanisms, such as: (a) barrel-stave model; (b) carpet model; and (c) toroidal model. A comprehensive review on oral antimicrobial peptides, their types and role in the oral cavity, including how these peptides are secreted and inhibit bacterial activities, has been reported elsewhere [28]. Cathelicidins and other antimicrobial peptides exhibited the potential of eliminating foreign pathogens through various processing pathways such as membrane disrupting activity, antiseptic activity, apoptosis, angiogenesis, wound healing, chemotaxis and immune modulation (both humoral and cellular components) [29]. Furthermore, these peptides target only pathogens, not human cells, due to the diversities within the biological membrane, including structure and composition. The broad spectrum antimicrobial property of cathelicidins is because of their ability to disrupt the bacterial cell membrane and ultimate death of bacteria. The three mechanisms proposed on how these peptides act on cell membranes include carpet model, barrel stave, and toroidal pore models [30,31]. The most lethal mode of action of an antimicrobial peptide is considered to be its interaction with the cytoplasmic membrane (Figure 2).

In the carpet model theory, peptides assemble themselves parallel to the surface of bacterial cell membrane, interacting electrostatically with phospholipid head groups on cell membrane surfaces. Hence, disintegration occurs due to the pressure created by the molecules of peptides and cell membrane in a detergent-like manner [24]. The barrel stave theory suggests that the formation of a pore in a manner that a single peptide is associated with the surface of the membrane at its hydrophobic region. The hydrophobic region is inserted further into the membrane. A number of peptide monomers self-aggregate and insert further into layer aligning each other perpendicular to the membrane to form a water filled pore. In the toroidal pore mechanism, the interactions of peptides with the phospholipid head groups, causing a fold in lipid bilayer rather than insertion and hydrophobic interactions [32,33].

In last decade, cathelicidins based drugs that are patented by different research groups. Zaiou et al. claimed that native LL-37 from human sweat have antimicrobial activity [34]. Shorter peptides with low hemolytic properties toward human blood cells were identified. In addition, enhanced antimicrobial activity, and the ability to synergize with the parent peptides were observed. Ståhle-bäckdahl and coworkers claimed an additional therapeutic potential of LL-37 and its derivatives for wound healing applications. These peptides facilitated the regenerative potential of the traumatized skin [35]. The Octoplus N.V. biopharmaceutical company (Amsterdam, Netherlands) reported two synthetic derivatives of LL-37 namely; peptide P10 (LAREYKKIVEKLKRWLRQVLRTLR-OH) for the treatment of infections related to atopic dermatitis and P60.4Ac (IGKEFKRIVERIKRFLRELVRPLR-OH) that can be used as a bioactive peptide layer for the prevention of bacterial growth and biofilm formation on the surfaces of biomaterials and metal implants [35,36]. Kosikowska et al. [37] reported antimicrobial peptides-based drugs and highlighted the importance of AMPs as a novel class of antibiotics (Table 1).

## 4. Importance of LL-37 in Oral Cavity

The environment contains an infinite number of microorganisms. For instance, bacteria are covering our skin, throat, gut, nasal cavity, ear, eyes, and oral cavity. Nature has protected the human body from the pathogenic bacteria by providing three lines of defense, including physiological, anatomical barrier i.e., skin, and certain immune cells to kill such pathogens. LL-37 has an immunomodulatory effects comprised of cellular and humoral components. The cellular component stimulates cells that play a crucial role in immunity and killing of foreign pathogens, for instance: neutrophils, macrophages, mast cells, dendritic cells, monocytes, and eosinophils. The humoral component (includes proteins, complement system and cytokines, cathelicidins) plays a different role. When considering the effects of cathelicidins on cells, for example, neutrophils, which play an important role during injury and inflammation, release in large number and are first to reach on such sites or infections. Neutrophils produce and express mediators, which include certain chemokines, cytokines, and fibrinogen and angiogenic factors. In addition, neutrophils stimulate the release of certain enzymes in the cytoplasm. Furthermore, they act as chemotactic agents, the source of prostaglandin, and leukotrienes, and are capable of generating reactive oxygen species. It has been reported that cathelicidins aid neutrophil in a number of ways; it increases the life span of neutrophils by expressing Bcl-XL proteins, and thus inhibiting early apoptosis of neutrophils [38]. LL-37 also increases chemotaxis activity and migration of neutrophils by inhibiting expression of surface receptors CXCR2. One of the principal mechanisms of innate immunity in which neutrophil extracellular traps (NETs) is formed (NETosis), cathelicidins aids to the formation of these NETs [39]. LL-37 affects other inflammatory cells activity as well, which includes monocyte or macrophages. In these cells it is also reported that they enhance the receptor expression on the site of injury, stimulate mediator release, aids in decreasing the endotoxin of *Neisseria meningitis*. In short, LL-37 stimulates these inflammatory cells and helps in the abolition of infections and inflammation, hence, playing a useful role in wound healing [3].

As discussed earlier, LL-37 also act as a broad-spectrum antibiotic in the human body. It was reported that this peptide provides essential role in innate response against *Mycobacterium tuberculosis*, as they stimulate alveolar macrophages as first line of defense against tuberculosis [40]. Another important role of cathelicidins is that it inhibits certain gastrointestinal (GI) disorders, such as ulcers, inflammation, and cancer, as these conditions commonly invade GI mucosa. Bacteria associated with gastritis and peptic ulcers are killed, which helps in repairing and angiogenesis of damaged tissues [41,42].

Furthermore, LL-37 is seen to be expressed in tongue and buccal mucosa, and also can be detected in GCF and saliva. While, inflamed gingival tissues has shown to have upregulated expression of LL-37, signifying its diagnostic activity in inflammatory periodontal disorders [43]. The role of LL-37 in saliva suggests its antimicrobial activity in the protection of tooth structure, which, in turn, can be correlated to resistance to caries. LL-37 presence in GCF contributes to the oral health status as it is seen to be associated with the severity of periodontal disease. For this reason, LL-37 antimicrobial quality makes them an excellent candidate for broad spectrum antimicrobial therapeutics, and plays a distinctive role in maintaining oral health [44].

In addition, LL-37 has an antiviral effect against certain viruses that includes influenza [45], *Herpes simplex*-1 [46], adenovirus, and human immuno-virus-1 (HIV-1) [47]. It has been reported that these peptides suppress the viruses by acting on their membrane envelopes and their protein capsules. On HIV, it works by disrupting the HIV-1 reverse transcriptase pathway through blocking its binding. In addition to antiviral effects of LL-37, there is antifungal role against important fungi such as *Candida albicans* that is present in our normal oral flora. Research using various types of cathelicidins, it is hypothesized that cathelicidins interact with the cell wall and generate reactive oxide species within fungi, leading to their fungicidal action [26].

## 5. Diagnostic Biomarker in Oral Health and Research

There are different approaches that are available to diagnose any clinical situation, according to the need, such as biopsy and bio-fluids analysis (saliva, blood, semen, sperm, cervico-vaginal secretions). Currently, human saliva plays an important role in the diagnostic sciences due to its beneficial features, such as non-invasive collection, cheap, easy to collect, and no need of any special clotting agents in contrast to blood testing. Saliva is rich in proteins and peptides, and has the ability to diagnose many human diseases [48,49,50,51]. Pakistan human salivary research group recently highlighted the importance of saliva as a diagnostic fluid for the detection of oral squamous cell carcinoma (OSCC) and the capability of salivary interleukins (IL-6 and IL-8) and tumor necrosis factor-α as a diagnostic biomarker for the detection of OSCC [52,53]. LL-37 is found in epithelial cells lining the oral cavity, tongue, buccal mucosa, inflamed gingival tissues, saliva, and GCF [54]. It’s concentration in saliva is approximately 0.14–3 μg/mL, and maintains protective activity against gingival lesions in addition to its significant role in wound healing [55]. Cathelicidins are well known for innate defensive barrier against various microbial pathogens, including gram negative and gram positive bacteria. Murakami et al. investigated the expression of messenger RNA of cathelicidins in the sialadenitis through reverse transcriptase-polymerase chain reaction and immunohistochemically staining. Cathelicidins protein expressions are upregulated in chronic sialedinitis compared to normal salivary glands and providing defense mechanisms in the salivary glands [14]. Moreover, cathelicidins has been broadly studied in relation to their immunomodulatory and antibacterial properties, whereas, salivary LL-37 is also being released by neutrophils in gingival crevicular fluid, salivary glands, and expressions of LL-37 indicates its role in the protection of tooth structure, oral mucosa, and enhances the production of immunoglobulins (IgA and IgG). Such measures control the bacterial checks for the prevention of biofilm and hence behaving as a natural antibiotic against dental caries [27]. Guo et al. tested children saliva in biofilm formation assay to evaluate the inhibitory effects of LL-37 on the biofilm that is produced by *Streptococcus mutans* and LL-37 interaction with epigallocatechin gallate (EGCG), which also has anti-infective property towards biofilm production. LL-37 enhanced the effects of EGCG on *Streptococcus mutans*, which, in turn, makes LL-37 as an anti-biofilm compound that can be potentially used for dental treatments [56].

LL-37 activates metalloproteinase via transactivation of the epidermal growth factor receptor (EGFR) receptor to elicit growth stimulatory properties, which helps in wound closure [57]. Kajiya et al. determined the effect of LL-37 on migration of human pulp cells by wound healing assay and immunoblotting. LL-37 plays a role in the enhancement of regeneration of pulp-dentin complex by activation of EGFR and c-Jun N-terminal kinase by the induction of heparin binding cell migration [58]. Furthermore, Tsai et al. performed competition assay and concluded that LL-37 has the property of reducing and inhibiting the infectivity of *Candida albicans* in the oral cavity by having effects on cell wall carbohydrates and can be used as a screening tool to check the involvement of fungal infections [59]. LL-37 seems to have role in the development of Papillon-Lefèvre syndrome. Eick et al. compared the levels of LL-37 in GCF, saliva, and neutrophil-derived enzymes, which revealed the fact that dysfunctional cathepsin C in patients of Papillon-Lefevre syndrome caused the deficit in immunomodulatory and antimicrobial functions of LL-37 in GCF, leading to severe periodontal disease. This occurs because of the lack of activation of cathepsin C in Papillon-Lefèvre syndrome, which ultimately results in deficit immunomodulatory and antimicrobial functions of LL-37 in gingiva that will allow bacteria such as *Aggregatibacter actinomycecomitans* to infect gingiva and periodontium to develop periodontal diseases. Hence, proving the ability of LL-37 as a diagnostic tool for inflammatory periodontal diseases [60]. In the same way, Montreekachon et al. investigated through methylthiazolyldiphenyl-tetrazolium bromide assay and real time-polymerase chain reaction in the gingival epithelial cells and reported the effects of LL-37 on Th1/Th2 cytokine expression. The results indicated the involvement of LL-37 in induction of IL-8 secretion and the recruitment of neutrophil at inflammatory sites in the periodontal tissues [61].

Several experimental studies have been conducted on evaluating the effects and outcomes of cathelicidins against bacteria (gram positive and gram negative), enveloped viruses, and fungi [17]. According to a study where unstimulated saliva was collected from children in correlation with their caries activity revealed that peptide based oral care products are protective in nature and provide defense against dental caries [62]. In patients that are diagnosed and recorded with periodontitis, the levels of cathelicidins were noted to be higher when compared to the healthy individuals.

Long term exposure to smoking leads to a reduction of cathelicidins, hence, the protective and antibacterial effects of the peptides are lost enhancing the probability of acquiring periodontitis [63]. The protective activity of cathelicidins was clearly demonstrated when a comparison of the levels was carried out using quantitative analysis (ELISA) between patients that were diagnosed with oral lichen planus and healthy individuals. Based on the study, higher levels were noted in severe forms of oral lichen planus (erosive) compared to reticular form, which, in turn, had higher levels when compared to their healthy counter-parts. These patients were periodically evaluated and as the lesion subsided clinically, the levels of the protective peptides also subsided [64]. This clearly states its anti-inflammatory and protective nature. Saliva collected from volunteers with healthy periodontium showed that this peptide has antimicrobial properties that are enabled in saliva. Thus, saliva acts as a mediator of its antimicrobial activity when tested against *E. coli*, but only in the presence of the organism *Porphyromonas gingivalis* proteases [65]. In vitro experiments have led to the conclusion that this could be a promising antimicrobial peptide supporting the host against livestock-associated methicillin-resistant *Staphylococcus aureus* (LA-MRSA), primarily because it is not influenced by common resistance genes [66]. Yi-jie Guo et al. experimented with EGCG, a constituent of tea catechins, which has potential of inhibiting not only microbial growth but on biofilm as well. Cathelicidins enhanced the activity of EGCG and opened a new horizon for potential anti-biofilm compounds [56]. Different sources of cathelicidins in the oral cavity were also evaluated, the quantitative study on the levels of cathelicidins using ELISA indicated lower levels in edentulous patients when compared to dentate patients. This has led to the theory that cathelicidins are released from the gingival tissues [25]. Keeping in view the above mentioned experimental studies, cathelicidins reinforce the protective and defensive antimicrobial properties, and play a vital role in the immunity.

## 6. Conclusions

Cathelicidins is a group of antimicrobial peptides that are secreted in the oral fluids, such as saliva, gingival crevicular fluid and can be used for diagnostic significance of oral health. The enhanced level of oral cathelicidins is associated with inflammatory conditions, such as gingivitis and immune disorders, such as oral lichen planus. In addition, the localized availability of cathelicidins may influence the immune response to oral microbial conditions, such as caries, periodontitis, and oral malignancies. Besides the diagnostic role, such an increase in the level of cathelicidins prevents infection (antimicrobial) and promotes wound healing of the effected tissues. Although the mechanism of action is not fully understood, the most likely mode of action of these antimicrobial peptides is through microbial membrane disruption.

## Figures and Tables

**Table 1:** Reported microbial inhibitory concentration (MIC) of different antimicrobial peptides sequences from LL-37 (adapted from Gallo and Murakami [37]).

Peptide	Sequence	*Staphylococcus aureus* (MIC (µM))	*Escherichia coli* (MIC (µM))	*Candida albicans* (MIC (µM))
LL-37	LLGDFFRKSKEKIGKEFKRIVQRIKDFLRNLVPRTES-OH	>64	64	20
RK31	RKSKEKIGKEFKRIVQRIKDFLRNLVPRTES-OH	16	8	4
KS30	KSKEKIGKEFKRIVQRIKDFLRNLVPRTES-OH	16	8	2
